# The prevalence of anti-factor VIII and anti-factor IX antibodies among patients with hemophilia in Rabat, Morocco: a single center experience

**DOI:** 10.11604/pamj.2022.41.126.29571

**Published:** 2022-02-14

**Authors:** Meryem Bouyadmar, Mohamed El khorassani, Maria El Kababri, Amina Kili, Laila Hessissen

**Affiliations:** 1 Department of Pediatrics, Cheikh Khalifa Ibn Zayed International University Hospital, Casablanca, Morocco,; 2 Hemophilia Treatment Center and Bleeding Disorders, Hematology-Oncology Unit, Children's Hospital Rabat, Mohamed V University, Rabat, Morocco

**Keywords:** Hemophilia, anti-factor VIII inhibitors, anti-factor IX inhibitors

## Abstract

The emergence of anti-factor VIII and anti-factor IX antibodies in hemophilia A or B is the most serious complication of hemophilia. We aim to expose through a series of patient's data, collected between 02/2009 and 02/2016 in the pediatric service of university hospital of Rabat, Morocco, the epidemiological and clinical characteristics of these patients, and to highlight the therapeutic difficulties encountered during their treatment. Out of 120 hemophiliac patients, we included 22 hemophiliac patients (18.33%, p<0.004) who developed an antibody, 21 patients with hemophilia A. Among the patients, 54.5% (n=12) exhibited moderate hemophilia, while 45.5% (n=10) had major hemophilia. The average age at diagnosis is estimated to 12±6.6 years. The circumstances of diagnosis were dominated by therapeutic inefficiency (63.64% (n=14)), then came dental extraction (9.09% (n=2)), preoperative assessment 22.73% (n=5) and hemophiliac arthropathy in a single case. The titration of antibodies in a 12-person sample ranged from 0.6 UB to 84 UB, of which (41.67% (n=5)) were low responders. The therapeutic treatment was based on fresh frozen plasma (54.55% (n=7)), recombinant activated factor VII (18.2% (n=4)), recombinant activated factor VII and PFC (18.2% (n=4)), and induction of immune tolerance. The occurrence of an inhibitory antibody represents the major residual complication of replacement therapy.

## Introduction

Hemophilia represents a bleeding disorder caused by X-linked genetic alterations in the production of coagulation factor. Hemophilia A, which interferes with the function of the factor VIII (FVIII) deficiency, is more common than hemophilia B, which involves factor IX, with a prevalence of one in 5,000 male live births compared to one in 30,000, respectively [1]. The development of antibodies anti-factor VIII and anti-factor IX is rare, they require constant replacement of the deficient factor. The disease severity in hemophilia is classified on the basis of the plasma level of FVIII or FIX activity. The severe form is defined as a factor level less 1 UI/mL, the moderate form as a factor level of 1UI/mL to 5UI/mL, and the mild form with a factor level greater than 5UI/mL and less than 40UI/mL [2]. Major recent advances in the treatment of hemophilia A include the emergence of extended half-life products, factor VIII orthologs, and gene therapy products. The factor replacement can be derived from plasma (pFVIII) and from voluntary donors or obtained by genetic engineering (recombinant FVIII or rFVIII). Most severe hemophiliacs receive prophylactic treatment with 2 to 3 injections per week.

Severe hemophilia cases become more resistant to the replacement therapy and require high doses of factor replacement [3]. The reported prevalence of factor inhibitors is less than 30% among patients with hemophilia A and less than 5% among patients with hemophilia B. Early studies consistently reported that the prevalence of inhibitors was 25% to 32%, although the prevalence may be as low as 12%, because some antibodies disappear over time. There are few reports regarding the prevalence of inhibitors in populations from the Eastern Mediterranean region. Therefore, the present study was performed to provide the first evaluation of FVIII and FIX inhibitors in Morocco.

## Methods

**Study population:** during seven years, going from 02/2009 to 02/2016, we collected 22 cases of hemophilia with anti-factor VIII or anti-factor IX inhibitors of which 21 cases are hemophiliac A and one case hemophiliac case B from 120 hemophiliac cases; including 95 hemophiliacs A and 25 hemophiliacs B, meeting the confirmatory/inclusion criteria for our study. All patients underwent a clinical examination, blood testing, and a short-standardized survey to collect their demographic and clinical data.

**Data collection:** the patients were recruited at the hematology oncology service of the children's hospital of Rabat University Hospital and during several hemophilia awareness days. Patients with a diagnosis of congenital hemophilia A or B were included if they fulfilled the following criteria. First, a diagnosis of development of anti-factor VIII or anti-factor IX inhibitors that was confirmed by the accredited central testing laboratory, and second, informed consent from the patient/guardian for participation. The farm return used for the post-processing of the collected data consisted of 4 sections, each of which consists of several answer/questions, most often of the closed type, single or multiple choice. The first component is to collect information about the patient, the patient's initials, the date of birth, the type of hemophilia and the degree of severity, social security coverage, family history, personal history (date of diagnosis of hemophilia and treatment received as well as the number and the type of bleeding episodes one year before the appearance of anti-factor VIII or anti-factor IX inhibitors). The second part provides data on the antibody, its date of appearance and the antibody titer, the circumstances of the diagnosis as well as the factor favoring the appearance of the anti-factor VIII or anti-factor IX inhibitors. The third part of the questionnaire is concerned with the therapeutic treatment of hemophilia patients followed in the hematology-oncology service of the children's hospital of the Rabat University Hospital. Thus, the questionnaire specified: i) treatment of bleeding episodes: factor VII activated or by prothrombotic complex or others as well as its mode of administration at home (home treatment) or at the hospital, and ii) induction of immune tolerance. The last part focused on the evolution of the disease after the appearance of the antibody; its disappearance or persistence, the number of bleeding episodes in the year following the appearance of the inhibitor and their location. The patients´ data were collected using a case report form and entered into a Microsoft Excel spreadsheet; these data were then reviewed by the study coordinators. After the coordinators had confirmed that the dataset was completed, the data were analyzed.

**Ethical considerations:** all patients provided their informed consent for participation and testing, and their demographic and laboratory data were stored in a password-protected repository.

**Statistical analysis:** was performed with R and python. The comparison between the different variables was made using the Chi-squared test for qualitative variables and the T-student test or Anova for quantitative variables. A p-value equal or less than 0.05 was considered statistically significant.

## Results

**Figure 1:**
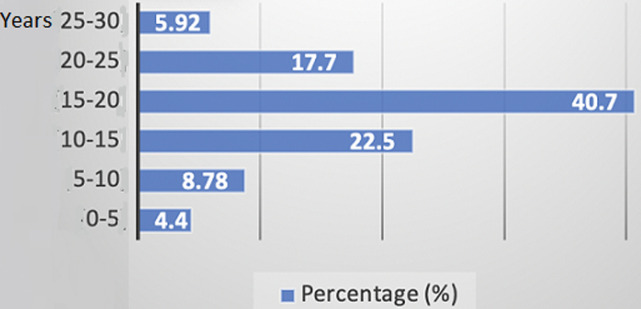
distribution of patients according to their age

**Figure 2:**
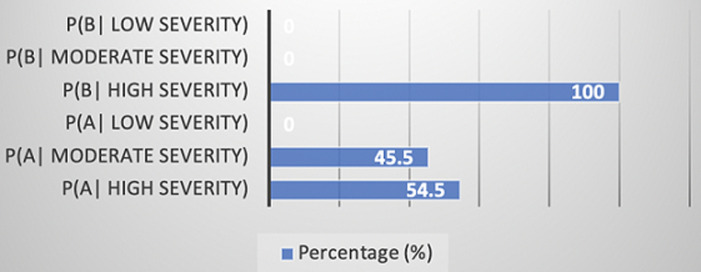
repartition of hemophilia A and B according to its severity

**Clinical characteristics:** all the 22 patients with a confirmed diagnosis of hemophilia were male (100%) and the age at the time of the anti-factor VIII or anti-factor IX inhibitors diagnosis in our series varies between 6 months and 27 years with an average age of 12 years and a standard deviation of 6.6 (Table 1). The average age of our patients was 15.55±5.96 years, with age extremes ranging from 3 years to 30 years. The 15-20 age range was the most represented in our series (p=0.008) (Figure 1). There was a predominance of patients from urban areas with a percentage of 77.27% (n=17) of cases (p=0.085). The family history of hemophilia is found in 7 cases (31.8%). Namely, 4 patients (18.18%) had similar hemophilia with anti-factor VIII or anti-factor IX inhibitors in their family, one single case had a brother with hemophilia without anti-factor VIII or anti-factor IX inhibitors, and 2 patients (9.09%) had brother died during circumcision. The hemophiliac type B patient had no family history. By coupling the statistical analysis of the type of hemophilia and the corresponding severity by adopting a simultaneous study of these two characteristics with a statistical analysis with two variables. Thus, we can calculate a conditional frequency of a property A knowing another property B, denoted P(A|B), as *P(A|B)= P ((A∩B))/P(A)*. The obtained results are shown in Figure 2 and their p-value is estimated to 0.032. The distribution of patients in this study according to the circumstances of anti-factor VIII or anti-factor IX inhibitors discovery are listed in Table 2.

**Table 1:** distribution of patients according to age groups at diagnosis

Age at diagnosis	Number of cases	Percentage (%)
6 months - 5 years	3	13.64
5 years - 10 years	4	18.18
10 years - 15 years	7	31.82
15 years - 20 years	6	27.27
20 years - 25 years	1	4.55
25 years - 30 years	1	4.55

**Table 2:** circumstances of the appearance of an inhibitor

Discovery circumstance	Number of cases	Percentage (%)
Hemophilic arthropathy	1	4.55
Preoperative assessment	5	22.73
Dental extraction	2	9.09
Non-effective therapeutic treatment	14	63.64

**Prevalence of anti-factor VIII or anti-factor IX inhibitors:** the two enabling factors that we detected in our study series were the age at which replacement therapy began, that is, an age less than 6 months (p<0.001), and the family history of hemophilia with antibody (p<0.001). Four patients (18.2%) were less than 6 months old at the beginning of replacement therapy, and 4 patients (18.2%) had family history of anti-factor VIII or anti-factor IX inhibitors. No promoting factor was revealed in the rest of the patients. Since before 2015, only the inhibition rate was measured in our hospital, we have only 12 patients in whom anti-factor VIII or anti-factor IX inhibitors titration is accessible; Results ranged from 0.6 UB to 84 UB with a standard deviation of 24.04 UB. 5 patients (41.67%) were considered as weak responders, while 7 patients (58.33%) were strong responders (with p=0.013). Three patients (13.64%) received replacement factor plasma, eight patients (36.36%) were on recombinant factor, while half of the cases (n=11) were under plasma and recombinant factors (with p<0.001).

**Treatment:** the therapeutic management of patients with anti-factor VIII or anti-factor IX inhibitors called for 54.55% (n=12) of cases with fresh frozen plasma; 18.2% (n=4) in addition to recombinant factor VII (Novoseven), fresh frozen plasma 18.2% (n=4) on Novoseven alone, and 9.1% (n=2) were, at the time of this study, under induction of immune tolerance treatment with p<0.001. One of them was a 13-year-old type A haemophiliac with a family history of a haemophiliac inhibitor brother. He was a strong responder and according to the immune tolerance induction register, high doses above 100 IU/kg/day were more effective than low doses, particularly in patients with an inhibitor titer above 10 Bethesda Units (BU). In January 2016, he had started a regimen of high doses of factor VIII at the rate of 100 IU/kg/d and the titration of inhibitor was tested every month and at least 24 hours after the last transfusion and which showed an oscillation between 4.3 BU and 20 BU (Table 3).

**Table 3:** the inhibitor titration of patient 5

Date	20/01/16	06/04/16	05/05/16	08/06/16	11/07/16
Titration in BU	16	20.16	13.28	4.3	7.36
Date	08/09/16	11/10/16	10/11/16	08/12/16	13/02/17
Titration in BU	10	16	14	10	9.6
Date	14/03/17				
Titration in BU	12.8				

## Discussion

This work reports the experience of the pediatric hematology-oncology service of the Rabat University Hospital, a service qualified as the first national center for the care of children with hemophilia. The aim of this work is to expose through a series of patients collected in our center the epidemiological and clinical characteristics and to highlight the therapeutic difficulties of these patients. Subsequent studies [4-5] carried out in several countries (Austria, USA, Finland, Germany, Sweden, Spain, Netherlands and Italy) show that in half of the patients the anti-factors VIII or IX appear before the age of 20 and around 70% before the age of 30. A study carried out at CHOP in 2010 showed that the hemophiliac population studied was of different ages between 4 months and 40 years of age [6]. However, there was a predominance of the age group between 2 and 10 years with a proportion of 43.56%. For hemophiliacs who developed these antibodies, they were of different ages ranging from 6 to 18 years with an average of 11 years, but the large part (33.33%) was between 9 and 10 years. This is consistent with our study where the age at diagnosis varied between birth and 27 years of age. Regarding the geographic distribution of this series of cases, the population was mainly concentrated in northwest Morocco, 21 cases, while only one patient came from the south (Agadir). No study has been conducted to this effect in Morocco. Hemophilia A is a risk factor for the development of an anti-factor VIII or anti-factor IX inhibitors. In the literature review, it was reported that approximately 25% of people with hemophilia A develop these types of complications while hemophiliacs B would be 5 to 10 times less exposed than hemophiliacs A [7]. Ninety five point four five percent (95.45%) of the population in our study was hemophiliacs A, and only 4.55% were type B which is in accordance with the literature. Thus, the prevalence of anti-factor VIII or anti-factor IX inhibitors in severe hemophilia A varies from 15 to 33% depending on the studies. In severe hemophilia B, it accounts for only 1 to 5% of cases [8]. Only 3 to 13% of moderate or minor hemophiliacs develop this complication [9]. It has been established that the existence of a family history of anti-factor VIII or anti-factor IX inhibitor increases by a factor of 2 the risk for a hemophiliac to develop these antibodies. This notion is confirmed in [10], with a relative risk of inhibitor in case of family history compared to hemophiliacs with relatives who have never developed anti-FVIII antibodies.

In our patients, the family history of hemophilia is found in 31.8% of cases, represented mainly by similar cases in siblings, i.e, 18.18% (n=4) of the cases. According to the results of the SIPPET study [11], treatment of severe hemophilia A with rFVIII is associated with an 87% higher incidence of anti-factor VIII or anti-factor IX inhibitors development than treatment with Factor VIII plasma containing Von *Willebrand* factor. In the present study, 13.64% (n=3) of our cases were treated before the appearance of a plasma anti-factor inhibitors, 36.36% (n=8) were on recombinant factor and half of the patients were substituted with both plasma and recombinant factor. It is therefore very difficult today to issue clear recommendations as to the type of FVIII (recombinant or of plasma origin) to be prescribed as first-line treatment in a severe hemophiliac A not previously treated (PUP). Defining the treatment of hemophiliac patients who have developed an anti-factor inhibitor is based on research and dosage of inhibitors of anti-hemophilia factors. They are classified as strong responders and weak responders according to the titration of anti-factor VIII or anti-factor IX inhibitors [12]. More than half of our cases (54.5%) received FFP as the only available product, 4 cases received FFP and recombinant factor VII while 4 patients were put on recombinant factor VII alone during bleeding episodes. Two patients in our study had received an induction of immune tolerance. One case is a hemophiliac A aged 13, with a family history of an inhibiting hemophiliac brother. It is a strong responder and according to the IITR register high dosages greater than 100 IU/kg/day are more effective than low doses, particularly in patients with an inhibitor titer greater than 10 BU. He had started in January 2016 a diet with high doses of factor VIII at a rate of 100 IU/kg/day and the inhibitor titration was tested every month and at least 24 hours after the last transfusion and which showed an oscillation between 4.3 BU and 20 BU. The second patient who started the immune tolerence induction (ITI) was a 19-year-old hemophiliac A with no family history who received a low-dose protocol of 50 IU/kg, 3 times a week. Clinically there were good evolutions. The two patients no longer bled after induction of immune tolerance.

## Conclusion

The occurrence of an inhibitory antibody represents the major residual complication of replacement therapy. In the event of the development of an inhibitor, anti-factor VIII or anti-factor IX, the treatment of hemorrhagic accidents calls upon activated coagulation factors, which are delicate to handle. The results of this study may be used as important tools to achieve a better understanding in the treatment of hemophilia patients. The presence of anti-factor VIII and anti-factor IX antibodies among patients might represent changes in therapeutic schemes of hemophilia patients and explain possible clinical complications in such patients. The incidence in hemophilia a carriers can be considered high when compared to other studies. The treatment with recombinant factor VIII must be evaluated for the implementation of measures that could avoid the development of factor VIII and IX inhibitors in patients who have not initiated treatment yet. This study is only the prelude to larger national surveys. Therefore, a national screening and counseling program for carriers, symptomatic patients, and asymptomatic persons will facilitate the early identification of cases and better management of patients with inhibitors.

### What is known about this topic

- *Hemophilia continues to be a disease of disastrous medical and social consequence for developing countries;*
- *Hemophilia (A or B depending on the type of deficiency, FVIII or FIX respectively) is an X-linked inherited bleeding disorder. The treatment of hemophilia uses coagulant factor concentrates, infused preventively in order to limit bleeding episodes*.

### What this study adds

- *Outline the epidemiological and clinical characteristics of a series of patients collected in the hemophilia treatment center at the children's hospital in Rabat and underline the therapeutic difficulties in the treatment phase;*
- *In the event of the development of an inhibitor, anti-factor VIII or anti-factor IX, the treatment of hemorrhagic accidents calls upon activated coagulation factors, which are delicate to handle. The administration of very high doses of antihemophilic factors aims to induce immune tolerance;*
- *The complexity of caring for hemophiliacs requires that they be monitored in reference centers by specialized multidisciplinary teams. This study is only the prelude to larger national surveys*.
